# Increasing Prevalence and High Risk of Associated Anomalies in Congenital Vertebral Defects: A Population-based Study

**DOI:** 10.1097/BPO.0000000000002124

**Published:** 2022-03-17

**Authors:** Susanna Heiskanen, Johanna Syvänen, Ilkka Helenius, Teemu Kemppainen, Eliisa Löyttyniemi, Mika Gissler, Arimatias Raitio

**Affiliations:** Departments of * Paediatric Surgery and Orthopaedics; ‡ Biostatistics, University of Turku and Turku University Hospital, Turku; † Department of Orthopaedics and Traumatology, University of Helsinki and Helsinki University Hospital; § Department of Information Services, Finnish Institute of Health and Welfare, Helsinki, Finland; ∥ Department of Molecular Medicine and Surgery, Academic Primary Health Care Centre, Karolinska Institute, Stockholm, Sweden

**Keywords:** congenital anomalies, congenital vertebral anomalies, pediatric spine, VACTERL

## Abstract

Congenital vertebral anomalies are a heterogeneous group of diagnoses, and studies on their epidemiology are sparse. Our aim was to investigate the national prevalence and mortality of these anomalies, and to identify associated anomalies.

**Methods:**

We conducted a population-based nationwide register study and identified all cases with congenital vertebral anomalies in the Finnish Register of Congenital Malformations from 1997 to 2016 including live births, stillbirths, and elective terminations of pregnancy because of major fetal anomalies. Cases were categorized based on the recorded diagnoses, associated major anomalies were analyzed, and prevalence and infant mortality were calculated.

**Results:**

We identified 255 cases of congenital vertebral anomalies. Of these, 92 (36%) were diagnosed with formation defects, 18 (7.1%) with segmentation defects, and 145 (57%) had mixed vertebral anomalies. Live birth prevalence was 1.89 per 10,000, and total prevalence was 2.20/10,000, with a significantly increasing trend over time (*P*<0.001). Overall infant mortality was 8.2% (18/219); 3.5% (3/86) in patients with formation defects, 5.6% (1/18) in segmentation defects, and 12.2% (14/115) in mixed vertebral anomalies (*P*=0.06). Co-occurring anomalies and syndromes were associated with increased mortality, *P*=0.006. Majority of the cases (82%) were associated with other major anomalies affecting most often the heart, limbs, and digestive system.

**Conclusions:**

In conclusion, the prevalence of congenital vertebral anomalies is increasing significantly in Finnish registers. Detailed and systematic examination is warranted in this patient population to identify underlying comorbidities as the majority of cases are associated with congenital major anomalies.

**Level of Evidence:**

Level III.

Congenital vertebral anomalies are a heterogenic group of diagnoses with an increased incidence of associated spinal and extraspinal anomalies.^1^–^4^ Prognosis and treatment vary from benign and less harmful to very difficult deformities and structural problems that include thoracic insufficiency syndrome, and spinal cord malfunction. It is estimated that prevalence of congenital vertebral malformations is ∼0.5 to 1 per 1000.^5^ However, total prevalence of these anomalies remains unknown as previous studies have not included stillbirths and elective terminations of pregnancies in their analyses, and population-based studies are lacking.

Vertebral anomalies may be an isolated finding, associate with other malformation or be part of an underlying chromosome abnormality or syndrome, like Klippel-Feil^6^ or vertebral defects, anal atresia, cardiac defects, tracheo-esophageal fistula, renal anomalies and limb abnormalities (VACTERL).^7^ Estimated frequency for associated malformations ranges from 30% to 70% depending on the type of vertebral anomaly.^1^,^4^ Cardiac system followed by urinary and gastrointestinal systems are most commonly affected.^4^ Over 90% of congenital vertebral anomalies are sporadic, however, 5% to 10% have genetic impact and are associated with chromosomal anomalies.^8^,^9^ Also, fetal alcohol syndrome has been reported to be associated with deformities of the cervical spine.^10^

The aim of this study was to assess the prevalence and mortality of different types of congenital vertebral anomalies in the Finnish population, and additionally to identify the most commonly associated anomalies and syndromes.

## METHODS

The Finnish Register of Congenital Malformations (FRM) contains data on all live births, stillbirths, and terminations of pregnancy because of severe fetal anomalies, and has been maintained by the Finnish Institute for Health and Welfare (THL) since 1963. It receives information from all maternity and pediatric hospitals, and also from other national health registers (the Medical Birth Register, the Register of Induced Abortions, the Care Register for Health Care, and the Register of Visual Impairment and National Hospital Discharge Register) and the Cause of Death register collected by Statistics Finland. More detailed description of the data collection protocol is provided in our previous publications.^11^–^13^ All recorded diagnoses in the register are evaluated, coded, classified, and double-checked by a medical geneticist and the coverage and data quality of the register have been considered good in several studies.^14^–^17^ If the diagnosis is unclear, more information (patient records, radiologic images and reports, autopsy results, laboratory findings) were requested from the hospitals by the medical geneticist.

The diagnoses in FRM are coded according to the International Statistical Classification of Diseases and Related Health Problems (ICD) by the World Health Organization (WHO)—both ninth and 10th revisions (ICD-9 and ICD-10) were used during our study period from 1997 to 2016. For our study, the register was searched with codes either 7561 (ICD-9) or Q76 (ICD-10) with all subcategories for congenital anomalies of spine and all matches were included in the study.

Cases were categorized based on the type of vertebral anomaly as proposed by McMaster and Ohtsuka:^18^ failures of vertebral formation (eg, hemivertebra), failures of vertebral segmentation (bony bar), and mixed vertebral anomalies (combination of different anomalies). Furthermore, cases were also classified as isolated, multiple congenital anomalies (MCAs, cases with other major anomalies) or syndromic (known syndrome, eg, trisomy) based on the manifestation of other comorbidities and EUROCAT syndrome guide.^19^ Concomitant major anomalies and syndromes were categorized based on EUROCAT classification system, and minor anomalies were excluded from the analyses.^20^ Prevalence of other major malformations was compared with reported prevalence in Finnish population including live births, stillbirths, and terminations of pregnancy because of fetal anomalies.^20^

The statistical analyses were performed using SAS System, version 9.4 for Windows (SAS Institute Inc., Cary, NC). Change in prevalence over the years was evaluated with linear regression and χ^2^ and the Fisher exact tests were utilized to analyze categorical variables. Relative risks along with 95% confidence intervals for associated anomalies compared with general prevalence of malformations were calculated. All tests were performed as 2-sided, with a significance level set at *P*<0.05. Birth prevalence and total prevalence are given per 10,000 births, and live birth prevalence is given per 10,000 live births as defined by EUROCAT.^20^

### Ethical Considerations

The approval of the Institutional Review Board at the Turku University Hospital was obtained before conducting this study and the Finnish Institute of Health and Welfare gave permission to use their register data in this study.

## RESULTS

**FIGURE 1:**
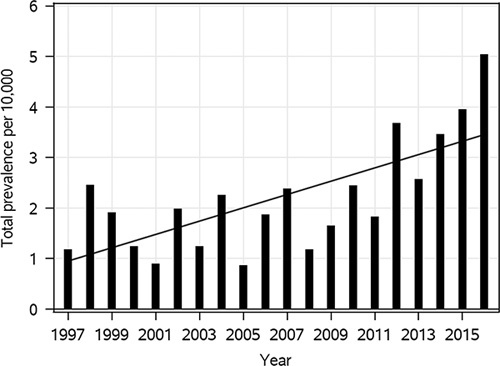
Increasing total prevalence of vertebral anomalies in Finland, *P*<0.001.

We identified 255 cases (120 males, 53.8%) with congenital vertebral anomalies from 1997 to 2016 in Finland. Of these, 92 (36%) were diagnosed with formation defects including 82 patients with hemivertebra, and 8 with butterfly vertebra. Segmentation defects were diagnosed in 18 (7.1%) patients, and 145 (57%) patients had mixed vertebral anomalies. There were 219 (85.9%) live births, 4 (1.6%) stillbirths, and 32 (12.5%) of the families opted for the termination of pregnancy. Total prevalence of vertebral anomalies including live births, stillbirths, and induced abortions because of anomalies was 2.20 per 10,000 births. Birth prevalence (live births and stillbirths) was 1.92 per 10,000 births and live birth prevalence was 1.89 per 10,000 live births. Total prevalence ranged from 0.86 to 5.03 per 10,000 births and the highest prevalence rates were recorded during the last 5 years of our study period. The prevalence of vertebral anomalies was increasing significantly during our study period, *P*<0.001 (Fig. 1). In subgroup analyses, only the prevalence of mixed vertebral anomalies was increasing significantly while moderately increasing trend was also seen in the prevalence of formation defects. In our cohort, the majority were born slightly preterm, on 37th to 38th week on average. Vaginal delivery (n=127, 58.0%) was more common than cesarean section (n=92, 42.0%). Cases with MCA or other syndrome were more often born through cesarean delivery than isolated cases: 46% versus 22% section rate, respectively (*P*=0.01).

The majority of cases 82.0% (209/255), and all of those with segmentation defects (100%) were associated with other major anomalies. An isolated spinal anomaly was only seen in 18.0% (46/255) and significantly more often among patients with formation defects or mixed vertebral anomalies, *P*<0.04 (Table 1). Most commonly affected organ systems were cardiac (34.9%), limbs (26.3%), and digestive system (25.9%) in that order (Table 2). There were 96 (37.6%) syndromic cases among which VACTERL was the most common (n=37) followed by chromosomal disorders (n=10), Goldenhar syndrome (n=8), Klippel-Feil syndrome (n=7), and spondylocostal dysostosis (n=7). Among terminated cases, there was only 1 (3.1%) isolated vertebral anomaly, 19 (59.4%) had MCA, and 12 (37.5%) were syndromic. The relative risk of having congenital anomalies in other organ systems compared with the whole Finnish population was elevated for all types of anomalies ranging from 10-fold to over 400-fold (Table 3).

**TABLE 1:** Demographics of Patients With Congenital Vertebral Anomalies

	Isolated Cases, n (%)	MCA, n (%)	Syndromic, n (%)
Formation defects (n=92)	(23.9) 22	(40.2) 37	(35.9) 33
Segmentation defects (n=18)	0	(22.2) 4	(77.8) 14
Mixed anomalies (n=145)	(16.6) 24	(49.7) 72	(33.8) 49
All (n=255)	(18.0) 46	(44.3) 113	(37.6) 96

MCA indicates multiple congenital anomalies.

**TABLE 2:** Incidence of Individual Concurrent Anomalies in Each Type of Vertebral Anomaly Organized by Affected Organ Systems

	Any, n (%)	Nervous System, n (%)	Heart, n (%)	Cleft, n (%)	Eye, n (%)	Ear, Face, and Neck, n (%)	Digestive System, n (%)	Respiratory, n (%)	Urinary, n (%)	Genital, n (%)	AWD, n (%)	Limb, n (%)	Chromosomal, n (%)	Other Anomalies/Syndromes, n (%)
Formation defects (n=92)	(76.1) 70	(15.2) 14	(31.5) 29	(4.3) 4	(2.1) 2	(3.3) 3	(22.8) 21	(4.3) 4	(14.1) 13	(5.4) 5	(2.2) 2	(20.7) 19	(7.6) 7	(46.7) 43
Segmentation defects (n=18)	(100) 18	(22.2) 4	(44.4) 8	0	(5.6) 1	(27.8) 5	(11.1) 2	0	(16.7) 3	(11.1) 2	(5.6) 1	(38.9) 7	0	(83.3) 15
Mixed anomalies (n=145)	(83.4) 121	(17.2) 25	(35.3) 52	(17.9) 26	(3.4) 5	(5.5) 8	(29.7) 43	(10.3) 15	(17.9) 26	(8.3) 12	(7.6) 11	(28.3) 41	(2.8) 4	(41.4) 60
All (n=255)	(82.0) 209	(16.9) 43	(34.9) 89	(11.8) 30	(3.1) 8	(6.3) 16	(25.9) 66	(7.5) 19	(16.5) 42	(7.5) 19	(5.5) 14	(26.3) 67	(4.3) 11	(46.3) 118

AWD indicates abdominal wall defect.

**TABLE 3:** Patients With Vertebral Anomalies Were Significantly More Likely Than General Population to Have Congenital Anomalies Affecting Other Organ Systems

	Any	Nervous System	Cardiac	Cleft	Eye	Ear, Face, and Neck	Digestive System	Respiratory	Urinary	Genital	AWD	Limb	Chromosomal
Formation defects (n=92)	18.68 (16.65-20.95)	31.36 (19.35-50.84)	15.67 (11.59-21.18)	45.45 (17.40-118.72)	9.69 (2.46-38.18)	30.15 (9.89-91.88)	87.75 (60.17-127.99)	246.08 (93.46-647.95)	32.93 (19.89-54.54)	344.58 (145.14-818.04)	30.54 (7.74-120.47)	36.21 (24.24-54.09)	18.08 (8.86-36.86)
All vertebral anomalies (n=255)	20.12 (18.98-21.33)	34.75 (26.43-45.70)	17.35 (14.66-20.52)	122.97 (87.42-172.99)	13.98 (7.06-27.69)	58.01 (35.98-93.52)	99.50 (80.60-122.85)	423.37 (268.97-666.40)	38.39 (29.08-50.68)	472.41 (299.36-745.48)	77.12 (46.14-128.91)	46.07 (37.46-56.67)	10.25 (5.75-18.28)

Values are given as relative risk and 95% confidence interval.

AWD indicates abdominal wall defect.

Overall infant mortality in patients with congenital vertebral anomalies was low, 8.2% (18/219). Patients with formation defects had the lowest infant mortality of 3.5% (3/86). In segmentation defects infant mortality was 5.6% (1/18), and 12.2% (14/115) in patients with mixed vertebral anomalies. Co-occurring anomalies and syndromes were significantly associated with higher infant mortality, *P*=0.006. There were no infant deaths in patients with isolated vertebral anomalies while MCA and syndromic cases were associated with respective infant mortality rates of 7.7% (7/91) and 13.3% (11/83). Infant mortality was also significantly higher among those born with cesarean section; 14.1% (13/92) versus 3.9% (5/127) with vaginal delivery, *P*=0.007.

## DISCUSSION

This population-based register study has revealed that the prevalence of congenital vertebral anomalies in Finland is increasing significantly and co-occurring congenital anomalies are seen in the great majority (82%) of this patient population. Regardless, vertebral anomalies are associated with low infant mortality, especially in the absence of concomitant anomalies in other organ systems.

Forrester and Merz^21^ observed a prevalence of 1.33 per 10,000 births for hemivertebrae in Hawaiian population. To the best of our knowledge, there are no population-based studies on the prevalence of other congenital vertebral anomalies. Previous studies have estimated the prevalence to be ∼0.5 to 1 per 1000,^5^,^8^,^18^ which is somewhat higher than the prevalence reported in the current study. Interestingly, we observed a significantly increasing trend in the prevalence of these anomalies in the Finnish population. We postulate that this may, at least in part, be explained by the advances and increased availability in both prenatal and postnatal imaging techniques leading to higher detection rates soon after birth. However, this increasing trend was observed especially during the last 5 years of our study period (2012 to 2016) and the major improvements in the imaging technologies (prenatal ultrasound, magnetic resonance imaging) have occurred well before this time period. Hence, it appears evident that the prevalence of congenital vertebral anomalies is increasing in Finland warranting further studies on the subject to identify potential risk factors. During our study period, the mean maternal age increased significantly, and the proportion of mothers over 35 years of age doubled.^22^ Advanced maternal age is a known risk factor for several congenital anomalies^23^,^24^ and could be one factor influencing the increasing prevalence. Also, prevalence of maternal diabetes increases with advancing maternal age,^22^ which further increases the risk of several congenital anomalies.^25^–^27^ Similar findings have not been reported previously. However, Lin et al observed an increasing prevalence of formation defects in their single center cohort of scoliosis patients in China.^28^

In accordance with previous publications, we also observed a high risk of associated major anomalies in patients with congenital vertebral anomalies. In our cohort, co-occurring anomalies were noted in 82% of patients while previous publications have estimated incidence rates of 30% to 70%.^1^,^4^ We postulate that this higher incidence reported here is likely a reflection of including stillborn and terminated cases. Passias et al^4^ reported cardiac, urinary, and digestive systems to be most commonly affected among patients with spinal abnormalities. Similarly, we also observed highest incidence of cardiac anomalies associated with vertebral anomalies and anomalies of urinary and digestive systems were frequently noted. However, abnormalities of limb(s) and nervous system were more common than previously reported.

The co-occurrence of vertebral defects, anorectal malformations, cardiac defects, tracheoesophageal fistula, renal anomalies, and limb abnormalities is identified as VACTERL association in cases where three or more of these anomalies are present.^7^,^29^ In our cohort, VACTERL association was the most common syndromic condition explaining significantly higher risk of anomalies in other organ systems related to this condition (cardiac, urinary, limb, and digestive system) observed in our study. In line with our findings, the association between vertebral anomalies and cleft lip and/or palate^30^ and abnormalities of the urogenital tract^31^ have also been reported earlier.

Even though vaginal delivery was more common than cesarean section in patients with congenital vertebral anomalies, the cesarean section rate of 42% far exceeds that of general population reported to be 15% in Finland during 1990 and 2006.^32^ Similar tendency has also been observed among other congenital anomalies even though the anomaly itself does not necessitate cesarean delivery.^11^,^12^ Interestingly, cesarean delivery was significantly associated with increased infant mortality in our cohort. However, we postulate that this may reflect the indications for the cesarean section rather than the increased risk of mortality associated with the cesarean delivery itself. This would be supported by the higher rate of cesarean delivery among complex cases in the present study.

Only 12.5% of the cases in our study were terminated because of fetal anomaly which is significantly lower than the termination rate of 51.5% for vertebral defects reported by a French-Canadian group.^33^ Prenatal detection of vertebral anomalies may be difficult unless associated with kyphoscoliosis.^34^ Even though most congenital vertebral anomalies are confirmed at approximately the 20th to 28th week of gestation,^34^ nearly one-third of the cases are diagnosed postnatally.^35^ As the fetal anomaly scan is performed between 18 and 22 weeks of pregnancy in Finland,^36^ it is possible that the number of occult vertebral lesions is higher than previously reported. Furthermore, the prenatal detection rate of these lesions was likely much lower during the early years of our study period because of less advanced technology. In addition to detection rate, there are regional differences related to culture, religion, and legislation affecting the termination rate for certain congenital anomalies.^11^

Previous studies have reported up to 50% mortality among patients with hemivertebrae presenting other associated anomalies.^21^,^35^,^37^ In contrast, Lemire et al^33^ reported only 6.7% neonatal mortality for patients with vertebral defects. Isolated cases of vertebral anomalies, although rare in this study, were not associated with mortality. As the majority of the cases in our cohort were associated with MCA or syndromes, the infant mortality rate of 8.2% reported here can be considered lower than expected based on previous literature.

The strength of study is that the register data stored in the FRM are both validated with high accuracy and a total population coverage.^13^,^38^ The main limitations are a relatively small sample size, especially among segmentation defects. Further, this study solely relies on the accuracy of register data. Also, our register data did not include information on the timing of the diagnosis preventing us making conclusions on prenatal versus postnatal diagnosis.

In conclusion, the prevalence of congenital vertebral anomalies has increased significantly in the FRM especially during the last 5 years our study period (1997 to 2016). The majority of cases are associated with other congenital comorbidities highlighting the importance of systematic examination and imaging. Screening for associated anomalies, especially cardiac defects, is recommended to optimize patient outcomes.
